# Highlighting the potential utility of MBP crystallization chaperone for *Arabidopsis* BIL1/BZR1 transcription factor-DNA complex

**DOI:** 10.1038/s41598-021-83532-2

**Published:** 2021-02-16

**Authors:** Shohei Nosaki, Tohru Terada, Akira Nakamura, Kei Hirabayashi, Yuqun Xu, Thi Bao Chau Bui, Takeshi Nakano, Masaru Tanokura, Takuya Miyakawa

**Affiliations:** 1 Department of Applied Biological Chemistry, Graduate School of Agricultural and Life Sciences, The University of Tokyo, Tokyo, 113-8657 Japan; 2 Department of Biotechnology, Graduate School of Agricultural and Life Sciences, The University of Tokyo, Bunkyo-ku, Tokyo 113-8657 Japan; 3 Graduate School of Biotsudies, Kyoto University, Sakyo-ku, Kyoto 606-8502 Japan; 4 Gene Discovery Research Group, RIKEN CSRS, Wako, Saitama 351-0198 Japan

**Keywords:** X-ray crystallography, X-ray crystallography, Computational biology and bioinformatics

## Abstract

The maltose-binding protein (MBP) fusion tag is one of the most commonly utilized crystallization chaperones for proteins of interest. Recently, this MBP-mediated crystallization technique was adapted to *Arabidopsis thaliana* (At) BRZ-INSENSITIVE-LONG (BIL1)/BRASSINAZOLE-RESISTANT (BZR1), a member of the plant-specific BZR TFs, and revealed the first structure of AtBIL1/BZR1 in complex with target DNA. However, it is unclear how the fused MBP affects the structural features of the AtBIL1/BZR1-DNA complex. In the present study, we highlight the potential utility of the MBP crystallization chaperone by comparing it with the crystallization of unfused AtBIL1/BZR1 in complex with DNA. Furthermore, we assessed the validity of the MBP-fused AtBIL1/BZR1-DNA structure by performing detailed dissection of crystal packings and molecular dynamics (MD) simulations with the removal of the MBP chaperone. Our MD simulations define the structural basis underlying the AtBIL1/BZR1-DNA assembly and DNA binding specificity by AtBIL1/BZR1. The methodology employed in this study, the combination of MBP-mediated crystallization and MD simulation, demonstrates promising capabilities in deciphering the protein-DNA recognition code.

## Introduction

Maltose-binding protein (MBP) is the most useful and successful crystallization chaperone for challenging proteins^1–4^, as MBP maintains the solubility of fusion proteins and is used as an affinity tag for protein purification^5–8^. Crystallization chaperones, including MBP, are also effective for determining novel crystal structures by molecular replacement (MR) methods using known protein structures as search templates^1^. The number of crystal structures solved by adopting the MBP fusion tag has increased in recent years (Supplementary Fig. 1)^3,4^. Moreover, this technique has been applied to various types of proteins, including nucleic acid binding proteins such as transcription factors (TFs)^9–13^.

BRZ-INSENSITIVE-LONG (BIL1)/BRASSINAZOLE-RESISTANT (BZR1) and its paralogs are key TFs in phytohormone brassinosteroid (BR) signaling, controlling thousands of genes, including growth-promoting genes and BR-synthesis genes, in *Arabidopsis thaliana*^14–20^. BIL1/BZR1 belongs to the plant-specific BZR TF family, in which members possess a highly conserved DNA binding domain (DBD) (Supplementary Fig. 2)^12,15,17^. BZR TFs preferentially recognize a G-box motif (C_1_A_2_C_3_G_4_T_5_G_6_)^12,18,19,21^, the universal *cis*-element in plants, and basic helix-loop-helix (bHLH) TFs are widely distributed in eukaryotes^22,23^. Although both DBDs of BZR and G-box-binding bHLH TFs harbor similar motif structures and common G-box-recognizing residues (Supplementary Fig. 2)^12,15^, BZR TFs do not strictly recognize C_1_A_2_ bases (complementary to T_5_G_6_ bases) of the G-box motif, as opposed to typical bHLH TFs^12,21^. In other words, BZR TFs have the potential to recognize one of the imperfect G-box variants, N_1_N_2_C_3_G_4_T_5_G_6_.

Recently, we resolved the first structure of *Arabidopsis thaliana* (At) BIL1/BZR1 DBD in complex with DNA by utilizing the MBP-mediated crystallization method^12^. AtBIL1/BZR1 adopts the noncanonical bHLH dimerization architecture, which consists of amino acid residues highly conserved in BZR TFs. Structural comparison of AtBIL1/BZR1 with typical bHLH TFs revealed molecular insight into the recognition of C_1_A_2_ bases by AtBIL1/BZR1 with lower specificity. Although MBP-mediated structural distortions have been reported to be very rare^3^, it is unclear whether the structure of MBP-fused AtBIL1/BZR1 in complex with DNA is the same as the native structure.

In the present study, we aimed to assess the validity of the reported crystal structure of the AtBIL1/BZR1-DNA complex chaperoned by MBP. Although the unfused AtBIL1/BZR1 DBD in complex with DNA was successfully crystallized, the poor quality of the X-ray diffraction data hindered the ability to resolve a structure that does not contain the MBP fusion tag. On the other hand, we conducted molecular dynamics (MD) simulations in an aqueous environment for the AtBIL1/BZR1-DNA complex derived from the crystal structures fused with MBP, which showed that there are no critical effects of MBP fusion or crystal packing on the AtBIL1/BZR1-DNA structure. In addition, our MD simulations clarify the structural basis governing the DNA binding specificity of AtBIL1/BZR1, which cannot be defined by crystal structures alone. The methodology employed in this study, the combination of MBP-mediated crystallization and MD simulation, demonstrates promising capabilities to precisely determine the molecular mechanism of DNA recognition by TFs or other DNA binding proteins.

## Results and discussion

### Crystallization and preliminary X-ray diffraction analysis of unfused AtBIL1/BZR1 DBD in complex with target DNA

**Figure 1:**
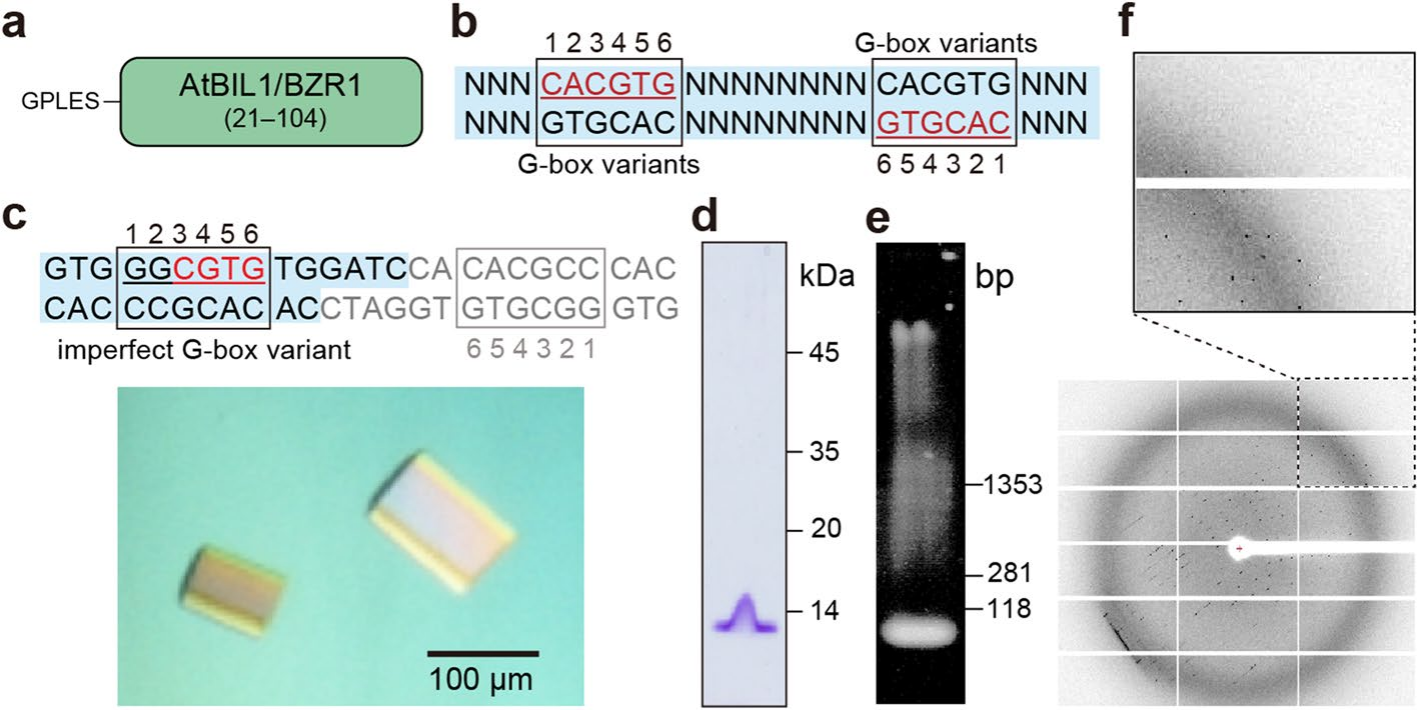
Crystallization and preliminary X-ray analysis of the unfused AtBIL1/BZR1 in complex with DNA. (**a**) Construction of the unfused AtBIL1/BZR1 used for crystallization. (**b**) DNA constructs successfully cocrystallized with unfused AtBIL1/BZR1. (**c**) Crystals of the unfused AtBIL1/BZR1 in complex with target DNA containing an imperfect G-box variant. The detailed sequence of the DNA is shown above the photograph. (**d**,**e**) SDS-PAGE analysis (**d**) and agarose gel electrophoresis analysis (**e**) of dissolved crystals. (**f**) X-ray diffraction image (3.0 Å at the edge) from a crystal.

To reveal the crystal structure of BZR TFs in complex with target DNA, we conducted crystal screening using the unfused DBD of AtBIL1/BZR1 (Fig. 1a). A few tens of DNA fragments containing the G-box motif or its variants were designed and used for cocrystallization experiments (Supplementary Fig. 3). Crystals were obtained when unfused AtBIL1/BZR1 was mixed with 26 base pair (bp) DNA fragments and palindromic DNA containing two G-box variants, as shown in Fig. 1b. Through the optimization of crystallization conditions and DNA constructs, we found a combination allowing us to obtain crystals suitable for X-ray diffraction analysis with high reproducibility. The optimized construct was a fragment of 26 bp DNA split into two with a protruding end and contained an imperfect G-box (G_1_G_2_C_3_G_4_T_5_G_6_) instead of a perfect G-box (Fig. 1c). The obtained crystals were confirmed to contain both unfused AtBIL1/BZR1 and the DNA fragment by SDS-PAGE and agarose gel electrophoresis analyses, respectively (Fig. 1d,e, Supplementary Fig. 4). These results suggested that the complex of unfused AtBIL1/BZR1 and target DNA was successfully cocrystallized. Subsequently, we collected X-ray diffraction data on a synchrotron radiation beamline but only obtained data with a resolution of > 3.1 Å (Fig. 1f). Although selenomethionine-containing AtBIL1/BZR1 mutants or an iodine-labeled DNA fragment was cocrystallized for phasing (Supplementary Fig. 5), all of these crystals produced poor diffraction data (> 5 Å resolution), resulting in the inability to resolve the structure of this complex.

### Strategy for the determination of the AtBIL1/BZR-DNA complex structure by MBP-mediated crystallization

**Figure 2:**
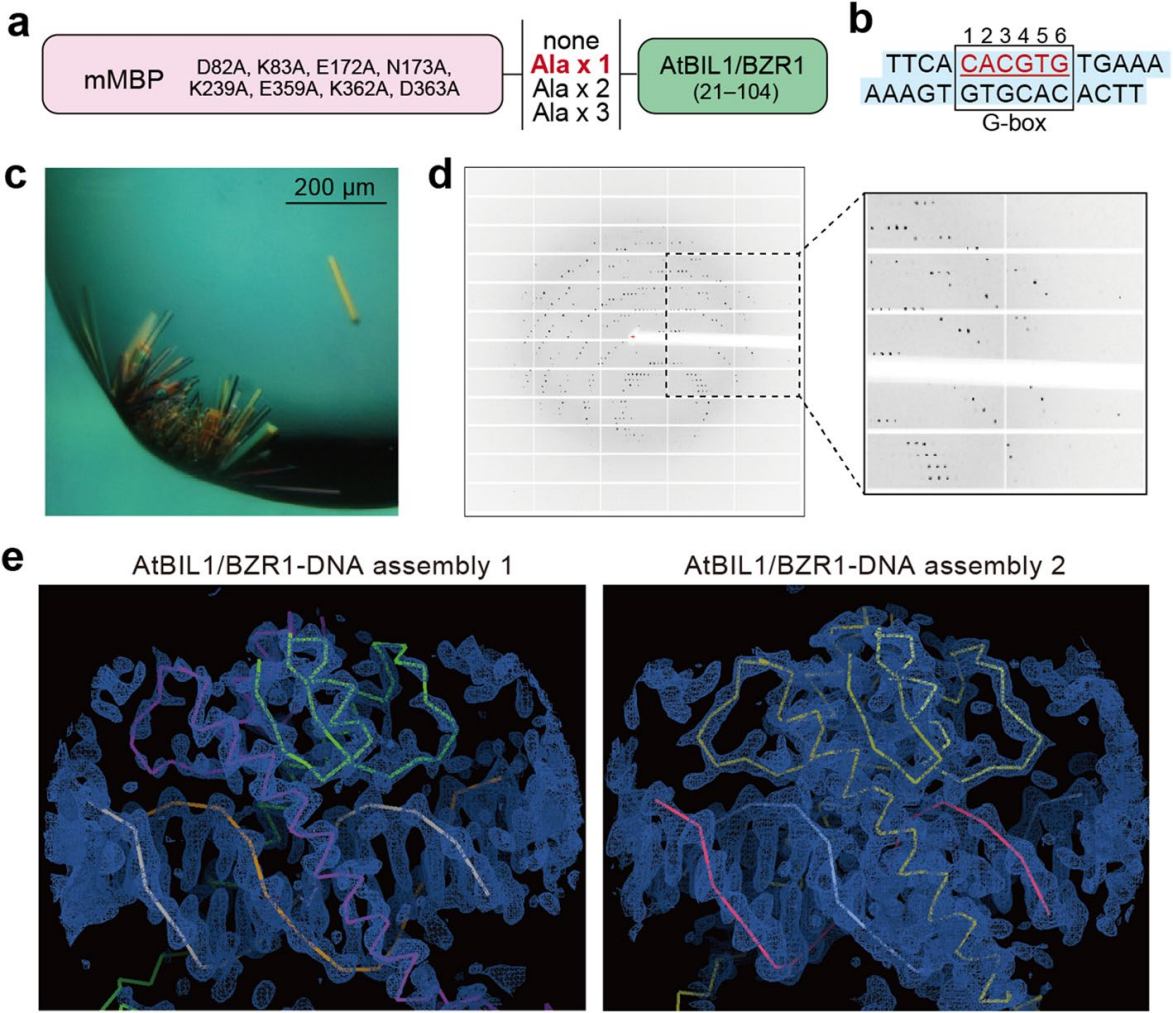
Crystallization and structure determination of MBP-fused AtBIL1/BZR1 in complex with DNA. (**a**) Mutant MBP (mMBP)-fused AtBIL1/BZR1 constructs for crystallization screening with different linker lengths. (**b**) DNA constructs successfully cocrystallized with mMBP-fused AtBIL1/BZR1 via one alanine linker. (**c**,**d**) Crystals (**c**) and an X-ray diffraction image (2.0 Å at the edge) from a crystal (**d**) of mMBP-fused AtBIL1/BZR1 in complex with the G-box-containing DNA. (**e**) Electron density map (2*F*_o_–*F*_c_) of AtBIL1/BZR1-DNA assemblies with contours at 1.5 σ (blue meshes) in the asymmetric unit of the reported structure (PDB ID: 5ZD4)^12^ depicted by the COOT program. The ribbons display the main-chain trace of two AtBIL1/BZR1 dimer-DNA complexes. Different colors represent different chains.

To obtain crystals suitable for high-resolution X-ray diffraction and phasing by the MR method, the MBP protein was fused to the N-terminus of AtBIL1/BZR1 DBD as a crystallization chaperone (Fig. 2a). Surface entropy reduction mutations were introduced into MBP to facilitate the formation of crystals, as utilized in previous studies^2,24–26^. Furthermore, we prepared four kinds of constructs with different linker lengths (0 to 3 Ala residue(s)). As with unfused AtBIL1/BZR1, dozens of DNA fragments containing the G-box motif or its variants were used for cocrystallization screening (Supplementary Fig. 3). Suitable crystals were obtained when only the mutant MBP (mMBP)-fused AtBIL1/BZR1 via one alanine linker and a palindromic 14 bp DNA with one nucleotide overhanging at the 3′ ends were mixed together (Fig. 2a–c). No crystals were obtained when 14 bp DNA variants and unfused AtBIL1/BZR1 were mixed, suggesting that MBP-mediated crystallization mainly contributed to the acquisition of high-quality crystals. X-ray diffraction data of the crystals were collected at a resolution of 2.17 Å (Fig. 2d)^12^. The structure of mMBP-Ala-AtBIL1/BZR1 in complex with the target DNA was solved by the MR technique using MBP as a template (Fig. 2e). In the asymmetric unit, there were four mMBP-Ala-AtBIL1/BZR1 chains and two double-stranded DNA fragments; two biological assemblies were composed of the AtBIL1/BZR1 homodimer and target DNA (Supplementary Fig. 6a). AtBIL1/BZR1-DNA assembly 2 was modeled from a higher quality electron density map and thus possessed lower *B*-factor values, whereas it was more difficult to model the side chain of AtBIL1/BZR1 in assembly 1 given the poorer electron density (Fig. 2e, Supplementary Fig. 6b).

### Structural dissection of crystal packing contacts between the MBP and AtBIL1/BZR1-DNA assemblies

**Figure 3:**
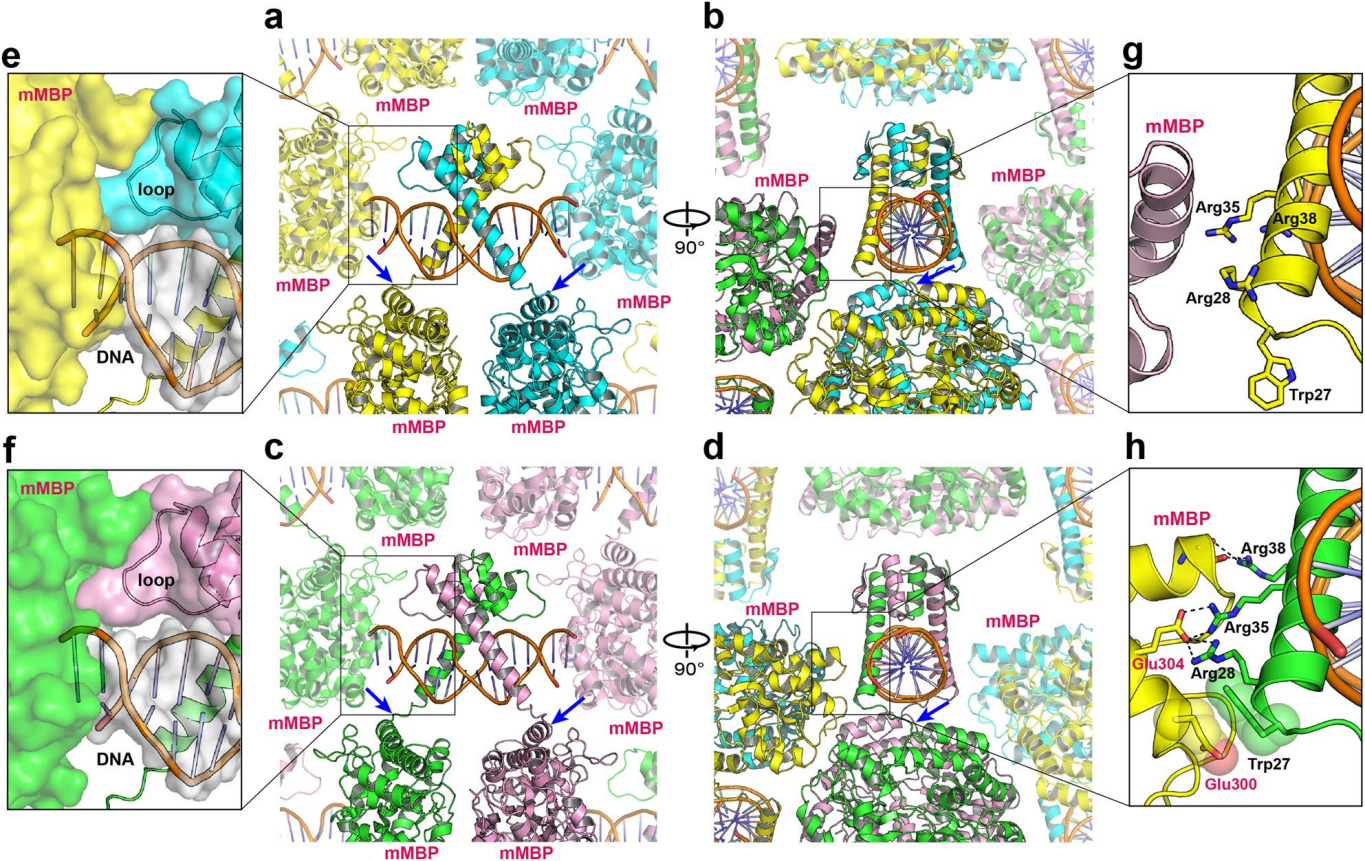
Crystal packing of MBP-fused AtBIL1/BZR1 in complex with DNA. (**a–d**) Front and side views of the crystal packing of AtBIL1/BZR1-DNA assemblies 1 (**a**,**b**) and 2 (**c**,**d**), depicted by PyMOL viewer. The boundaries between the C-terminus of mutant MBP (mMBP) and the N-terminus of AtBIL1/BZR1 are indicated with blue arrows. (**e**,**f**) Close-up views of the packing between the mMBP (yellow or green surface model) and DNA ends (white surface model) or AtBIL1/BZR1 loops (cyan or magenta surface model). (**g**,**h**) Close-up views of the spatial positioning of mMBP and DNA recognition helices of BIL1/BZR1. Hydrogen bonds and salt bridges are indicated by dashed lines. The residues involved in van der Waals interactions are shown as sphere models.

Some crystal packing contacts were observed in the MBP-fused AtBIL1/BZR1 DBD complexed with DNA. The C-terminus of MBP and the N-terminus of AtBIL1/BZR1 were connected without adopting a specific secondary structure, suggesting that MBP fusion does not directly constrain the structures of AtBIL1/BZR1 (Fig. 3a–d). The two variants of the AtBIL1/BZR1-DNA assemblies were surrounded only by MBP proteins. Although the protruding ends of DNA were embedded into the MBP surfaces (Fig. 3e,f), there were no crystal packing contacts in the DNA region bound to AtBIL1/BZR1 (Fig. 3a–d). The loop region of AtBIL1/BZR1 also contacted the MBP proteins via van der Waals interactions, which were formed in a similar manner in both assemblies (Fig. 3e,f). In addition, the crystal packing of MBP and DNA recognition helices of AtBIL1/BZR1 were distinct in the two assemblies because of different spatial positionings of the MBP and AtBIL1/BZR1-DNA assemblies (Fig. 3b,d,g,h). The side chains of three arginine residues (Arg28, Arg35 and Arg38) from AtBIL1/BZR1 in assembly 2 formed salt bridges with a glutamic acid residue (Glu304) or a hydrogen bond with the main chain of MBP (Fig. 3h), whereas no apparent interactions were observed between MBP and the DNA recognition helices of AtBIL1/BZR1 in assembly 1 (Fig. 3g). In addition, Trp27 of AtBIL1/BZR1 in assembly 2 contacted another glutamic acid residue (Glu300) of MBP via van der Waals interactions (Fig. 3h). Although the two assemblies had different crystal packing arrangements, there were few differences in the overall structures of the DNA recognition helices of the two assemblies, indicating that crystal packing contacts with MBP appear not to directly distort DNA recognition helices (Fig. 3g,h). Consequently, we infer that the crystal packing contacts of DNA recognition helices may reinforce the fixed relative position of AtBIL1/BZR1-DNA assembly 2 with respect to the MBP crystallization chaperone, resulting in a lower *B*-factor, a measure of local mobility in the molecule, than that of assembly 1.

### MD simulations of the AtBIL1/BZR-DNA complex with the removal of the MBP chaperone

**Figure 4:**
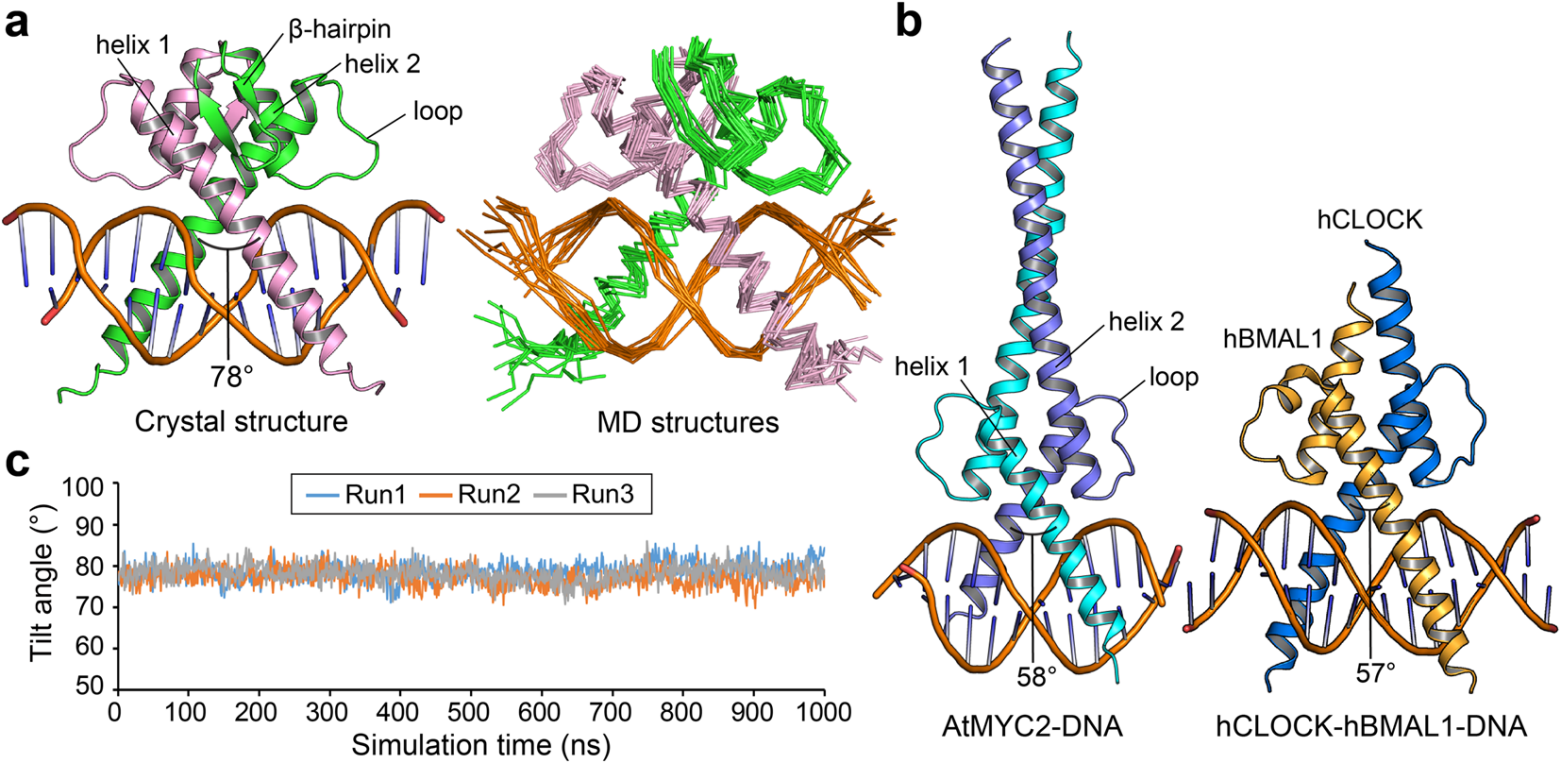
MD simulations for the AtBIL1/BZR1-DNA complex. (**a**) The crystal structure and MD structures (every 100 ns (ns) up to 1000 ns) of the AtBIL1/BZR1-DNA complex (PDB ID: 5ZD4, chains C, D, G and H, assembly 2). The tilt angle between the DNA recognition helices is shown on each crystal structure. (**b**) Crystal structures of the AtMYC2-DNA complex (PDB ID: 5GNJ, chains A–D) and hBAL1-hCLOCK-DNA complex (PDB ID: 4H10, chains A–D). (**c**) Tilt angles between DNA recognition helices of AtBIL1/BZR-DNA every 1 ns up to 1000 ns (three independent runs). The MD structures (**a**) correspond to the results of Run 3.

Despite multiple attempts, the structure of the unfused BIL1/BZR1-DNA complex at approximately 3.1 Å resolution could not be resolved by the MR method using derivatives from the MBP-fused BIL1/BZR1-DNA complex, which may be due to crystal twinning (twin fraction, 0.244). Since we suspected that there was a significant difference in the two AtBIL1/BZR1 structures, for more reliable structural consideration, we conducted MD simulation using assembly 2 of the crystal structure of AtBIL1/BZR1-DNA from which MBP chaperones had been removed. Root-mean-square deviations (RMSDs) from the crystal structure were calculated after aligning the protein Cα atoms of each snapshot structure to those of the crystal structure, were 1.88 ± 0.31 Å and 2.41 ± 0.40 Å for the protein Cα and DNA phosphorus atoms, respectively. These results indicate that the complex structure was stably maintained during the MD simulation runs. Specifically, AtBIL1/BZR1 is characterized by the β-hairpin structure following helix 2, which is shorter than that in typical bHLH TFs, including *A. thaliana* MYC2 and *Homo sapiens* BMAL1-CLOCK (Fig. 4a,b)^12,27,28^. This β-hairpin structure of AtBIL1/BZR1 was also retained for the entire simulation time (1 μs), covering helix 2 of the same chain and helix 1 of another chain (Fig. 4a, Supplementary Fig. 7). The two helices and β-hairpin composed a noncanonical bHLH dimerization architecture, which gave AtBIL1/BZR1 a larger tilt angle between the DNA recognition helices (78°) than those of any bHLH TFs whose structures have been reported (50°‒65°) (Fig. 4a,b)^12^. In the MD structures as well as in the crystal structure, AtBIL1/BZR1 remained at a larger tilt angle between helices (78.0° ± 2.3°) than that of bHLH TFs (Fig. 4c). Therefore, our MD simulation demonstrated that the distinct dimerization architecture of AtBIL1/BZR1 was not due to a structural distortion caused by MBP-mediated crystal packing but directly reflected a characteristic amino acid sequence that is highly conserved in plant-specific BZR TFs (Supplementary Fig. 2).

### Defining the C_1_A_2_ base recognition mode of BZR TFs

**Figure 5:**
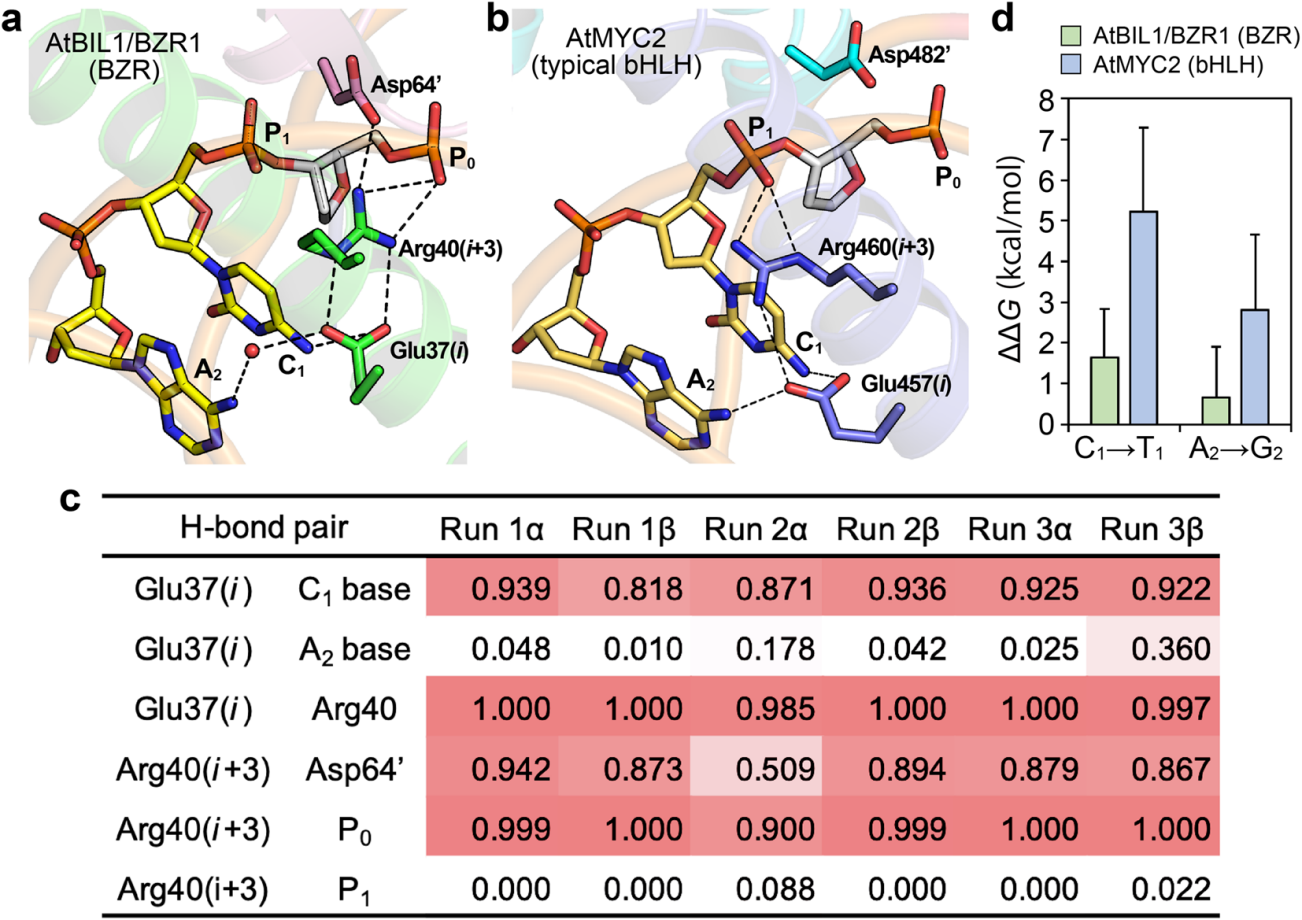
The C_1_A_2_ base recognition mode of BZR TFs is distinct from that of typical bHLH TFs. (**a**,**b**) The essential hydrogen-bonding networks for C_1_A_2_ base recognition by the AtBIL1/BZR1-DNA complex (**a**) and AtMYC2-DNA complex (**b**), which are observed in the crystal structures. The residues with or without a prime mark (’) belong to different chains. ‘P_N_’ represents a phosphate group at position N. Dashed lines and a red sphere represent hydrogen bonds (including salt bridges) and a water molecule, respectively. (**c**) Ratios of hydrogen bond (H-bond) formation in MD structures of the AtBIL1/BZR1-DNA complex. The results of both chains α and β, which correspond to chains C and D of AtBIL1/BZR1 (PDB ID: 5ZD4), respectively, are shown for 3 independent runs. Colors closer to red indicate a higher ratio. (**d**) The binding free-energy differences (ΔΔ*G*) between different nucleobases (C_1_ to T_1_ and A_2_ to G_2_) in complex with AtBIL1/BZR1 or AtMYC2. Data are the means + standard deviations (SDs, n = 6 independent runs).

AtBIL1/BZR1 has been found to recognize C_1_A_2_ bases (complementary to T_5_G_6_) in the G-box motif (C_1_A_2_C_3_G_4_T_5_G_6_) with a lower specificity than that found in typical bHLH TFs. A comparison of crystal structures of AtBIL1/BZR1 and typical G-box-binding bHLH TFs suggested that there is a difference in the relative orientation of the key glutamic acid residues Glu(*i*), which are essential for recognizing C_1_A_2_ bases (Fig. 5a,b, Supplementary Fig. 8). In typical bHLH TFs, including AtMYC2, the Glu(*i*) residues directly interacted with both the C_1_ and A_2_ bases via hydrogen bonds, which were sustained by hydrogen-bonding networks with conserved arginine residues Arg(*i* + 3) and DNA phosphate groups at position 1 (P_1_) (Fig. 5b, Supplementary Fig. 8)^12,27–29^. On the other hand, Glu37(*i*) of AtBIL1/BZR1 indirectly recognized the A_2_ base through a water-mediated hydrogen bond because of the distinct orientation of Arg40(*i* + 3), which interacted with the highly conserved Asp64 residue on the loop and P_0_ instead of P_1_ (Fig. 5a).

To evaluate the validity of the C_1_A_2_ bases and phosphate recognition modes found in the crystal structure of AtBIL1/BZR1-DNA, we investigated the formation of each hydrogen bond pair from MD structures (Fig. 5c). Arg40(*i* + 3) continued to interact with P_0_ (at least 90%) and Asp64 (more than 50%) but not with P_1_ (less than 10%). Through a tight salt bridge with Arg(*i* + 3), Glu37(*i*) remained distant from the A_2_ base, resulting in a weaker interaction with the A_2_ base. In addition, our further dissection combined with MD simulations revealed that the side chains of Glu37(*i*) and Arg40(*i* + 3) formed tight hydrogen bonds (salt bridges) on the same plane where the C_1_ base was not located. This observation suggested that the hydrogen bond between Glu37(*i*) of AtBIL1/BZR1 and the C_1_ base was relatively weak (Fig. 5a, Supplementary Fig. 9). Moreover, we calculated differences in the binding free energy (ΔΔ*G*) when the C_1_A_2_ bases were substituted with T_1_A_2_ and C_1_G_2_ in complex with AtBIL1/BZR1 or AtMYC2 by the free-energy perturbation method (Fig. 5d). The ΔΔ*G* values for the AtBIL1/BZR1-DNA complex indicate that substitutions with T_1_A_2_ and C_1_G_2_ only slightly reduced the affinity of AtBIL1/BZR1. In contrast, large positive ΔΔ*G* values were observed for the AtMYC2-DNA complex, indicating that the affinity of AtMYC2 was greatly reduced by the substitutions. These simulation results are in agreement with previously reported studies on the DNA binding specificity of BZR TFs and typical bHLH TFs^12,21^, thereby also strongly supporting the distinct C_1_A_2_ base recognition modes between AtBIL1/BZR1 and AtMYC2 observed in their crystal structures (Fig. 5a,b). The larger tilt angle between DNA recognition helices of AtBIL1/BZR1 changed the relative positions among Glu37(*i*), Arg40(*i* + 3), and Asp64 on the loop, and DNA nucleobases and phosphate groups, which was different from the configuration observed in typical bHLH TFs. The C_1_A_2_ base recognition mode of AtBIL1/BZR1 was postulated to be achieved by the larger tilt angle between helices. Consequently, our MD simulations defined the structural mechanism for a weaker interaction between the C_1_A_2_ bases and the AtBIL1/BZR1 DBD, which is highly conserved in BZR TFs.

## Conclusions

The MBP crystallization chaperone has been applied to reveal the crystal structure of the AtBIL1/BZR1 DBD in complex with target DNA, which has not been determined using the unfused BIL1/BZR1 construct. The length, position of a core-binding site, and identity of the termini of the DNA molecule play critical roles in crystallization with AtBIL1/BZR1 (even in the unfused construct)^30,31^. Together, investigating the linker length between MBP and the target protein also greatly contributed to the successful crystallization of the MBP-fused BIL1/BZR1-DNA complex, as reported in a recent study^4^. Furthermore, the technique of MBP-mediated crystallization enabled us to simply solve the crystallographic phase problem by the MR method using the MBP structure as a template and even to determine the high-resolution structure. Other than AtBIL1/BZR1, four structures of nucleic acid-bound proteins have been revealed by MBP fusion crystallographic systems^9–11,13^. Moreover, there are no successful structural analyses of nucleic acid-bound proteins with other fusion crystallographic systems including thioredoxin (Trx)- or glutathione *S*-transferase (GST)-fusion. Furthermore, the AtBIL1/BZR1-DNA complex is the first successful example using an MBP crystal chaperone aimed at deciphering DNA binding specificity. Since water plays an important role in both the specificity and affinity of protein-DNA interactions and a high-resolution structure allows the observation of water molecules^32–34^, adapting MBP-mediated crystallization to protein-DNA complexes is effective for understanding the structural basis for DNA recognition by proteins such as AtBIL1/BZR1. However, there is a possibility that MBP fusion or crystal packing would cause structural distortion of the protein of interest. Since the crystal structure of the unfused AtBIL1/BZR-DNA complex was unsuccessfully resolved for unclear reasons including crystal twinning, we conducted MD simulations in an aqueous environment using the AtBIL1/BZR1-DNA complex derived from the MBP-fused crystal structure. MD simulation has been used as a powerful approach for dissecting DNA binding specificity by various types of TFs^35–40^. Furthermore, the present study shows that MD simulation is also a promising approach to estimate the validity of MBP-fused crystal structures instead of solving corresponding unfused structures with difficulty. The strategy adopted in this study, which combines MBP-mediated crystallization and MD simulations, is shown to be capable of deciphering the protein-DNA recognition code of interest.

## Materials and methods

### Sequence alignments

CLUSTAL OMEGA^41^ was used for multiple sequence alignments among BZR TFs or typical bHLH TFs using default parameters, and the results were displayed by ESPript 3.0^42^. Aligned sequences included AtBIL1/BZR1 (At1g75080), AtBES1 (At1g19350), AtBEH1 (At3g50750), AtBEH2 (At4g36780), AtBEH3 (At4g18890) and AtBEH4 (At1g78700) from *A. thaliana*, OsBZR1 from *Oryza sativa* (LOC_Os07g39220), XP_016508570 from *Nicotiana tabacum* and KK1_013025 from *Cajanus cajan* for BZR TFs, and AtMYC2 from *A. thaliana*, MYC, MAD, MAX, BMAL1 and CLOCK from *H. sapiens* and PHO4 from yeast for the typical bHLH TFs.

### Expression and purification of the unfused BIL1/BZR1 DBD

Codon-optimized *Arabidopsis thaliana* BIL1/BZR1 (21A–104R) was cloned into pGEX-6P-3 (GE Healthcare) with an N-terminal glutathione S-transferase (GST) tag and a human rhinovirus (HRV) 3C protease cleavage site. Isopropyl β-D-1-thiogalactopyranoside (IPTG)-induced overexpression was performed for 2 h at 37 °C. Cells were harvested by centrifugation at 5000 rpm for 15 min and stored at − 80 °C until use. The harvested cells containing GST-fused AtBIL1/BZR1 were resuspended in buffer A (20 mM Tris–HCl at pH 7.5, 1.0 M NaCl, 1 mM DTT and 5% glycerol) and were then lysed by sonication. The cell debris was removed by centrifugation at 40,000 × g for 30 min. The supernatant fractions were then applied to Glutathione Sepharose 4B resin (GE Healthcare). After washing with buffer A, the HRV 3C protease was added to remove the GST tag, and the unfused protein was then eluted with buffer A. The eluate of unfused AtBIL1/BZR1 was concentrated with a Vivaspin 15R device (10,000 MWCO Hydrosart, Sartorius) and further purified by loading onto a HiLoad 26/60 Superdex 75 pg column (GE Healthcare) against buffer B (20 mM Tris–HCl at pH 7.5, 0.5 M NaCl, 1 mM DTT and 5% glycerol). The purified protein was concentrated to 1.0 mM in preparation for cocrystallization with DNA.

### Crystallization and preliminary X-ray diffraction analysis of the unfused BIL1/BZR1 DBD in complex with DNA

The DNA fragments for cocrystallization were dissolved in buffer B (20 mM Tris-HCl at pH 7.5, 100 mM NaCl, and 1 mM EDTA) and then added in 1.5-fold molar excess to unfused AtBIL1/BZR1 in buffer C (20 mM Tris-HCl at pH 7.5, 150 mM KCl, 1 mM DTT and 5% glycerol). The mixture was concentrated until the DNA concentration was 1.0–1.4 mg/ml. Crystals of the unfused AtBIL1/BZR1-DNA complex above were obtained using the sitting-drop vapor diffusion method with the reservoir solution consisting of 50 mM MES-NaOH at pH 5.6, 200 mM ammonium acetate, 10 mM calcium chloride and 10% (w/v) polyethylene glycol (PEG) 4000 at 20 °C. All crystals were transferred to the reservoir solution containing 26% ethylene glycol as a cryoprotectant and flash-cooled at 95 K with annealing. X-ray diffraction data were collected on beamline NE-3A at the Photon Factory (Tsukuba, Japan) using a Pilatus-2 M detector. All X-ray diffraction data were integrated and scaled using the programs XDS^43^ and AIMLESS^44^, respectively.

### Structural dissections and comparisons

The electron density maps were displayed using the program COOT (Crystallographic Object-Oriented Toolkit)^45^ (Ver. 0.9 EL). Structural dissections and comparisons were conducted, and the images were depicted using the molecular graphics system PyMOL (Ver. 2.4, Schrodinger, LLC).

### MD simulations

The coordinates of the AtBIL1/BZR1 homodimer (residues 21–88) and the DNA were extracted from those of assembly 2 of the crystal structure (PDB ID: 5ZD4). The N- and C-termini of the AtBIL1/BZR1 chains were capped with acetyl and *N*-methyl groups, respectively. The AtBIL1/BZR1-DNA complex was solvated in a cubic water box with an edge length of approximately 82 Å, and potassium ions were placed around the complex to neutralize the system. Amber ff14SB force field parameters^46^ were used for proteins, OL15 parameters^47–49^ were used for DNA, and the TIP3P model^50^ was used for water. After energy minimization, each system was equilibrated at 300 K and 1.0 × 10^5^ Pa with a 1-ns MD simulation. Position restraints were imposed on the nonhydrogen atoms of the protein and the DNA. In addition, distance restraints were imposed between Oε1 of Glu37 and Nε of Arg40, between Oε2 of Glu37 and Nη2 of Arg40, between OP2 of A_0_ and Nη2 of Arg40, and between N7 of G_4_ and Nη2 of Arg41′. The position restraining force was gradually weakened during the simulation. Subsequently, a 100-ns MD simulation was performed with distance restraints, of which the force constant was gradually reduced during the simulation. Finally, a 1-μs MD simulation was performed without restraints. This series of MD simulations was repeated three times with different initial velocities. In all MD simulations, the temperature was controlled by the velocity-rescaling method^51^, and the pressure was controlled by the Berendsen weak coupling method^52^. Bond lengths involving hydrogen atoms were constrained using the LINCS algorithm^53^ to allow the use of a large time step (2 fs). Electrostatic interactions were calculated with the particle mesh Ewald method^54,55^. MD simulations were performed with Gromacs 2018^56^, with coordinates recorded every 10 ps. MD simulations for the AtMYC2-DNA complex were conducted in the same manner except the following: The coordinates were obtained from the PDB (PDB ID: 5GNJ), and the complex was immersed in a cubic water box with an edge length of approximately 127 Å. Distance restraints were imposed between N4 of C_1_ and Oε1 of Glu457, between N6 of A_2_ and Oε2 of Glu457, between Oε2 of Glu457 and Nη1 of Arg460, between OP2 of C1 and Nε of Arg460, between OP2 of C1 and Nη2 of Arg460, and between N7 of G4 and Nη1 of Arg461’.

Binding free-energy differences between different DNA sequences were calculated by the free-energy perturbation method. A purine base of the original DNA (referred to as DNA_1_) was chemically transformed into the other type of purine base in 21 steps through 19 intermediate states. At the same time, the pyrimidine base that forms a base pair with the purine base was also transformed into the other type of pyrimidine base to give an altered DNA sequence (referred to as DNA_2_). In each step, a 2-ns MD simulation was performed, and the free-energy differences between the adjacent states were calculated from the last 1-ns MD trajectory using the Bennett acceptance ratio method^57^. The sum of all steps gives the free energy difference, Δ*G*, caused by the change in the bases. The free energy differences were calculated for the DNA alone [Δ*G*(DNA_1→2_)] and the protein–DNA complex [Δ*G*(complex_1→2_)]. Let Δ*G*_bind,*i*_ be the binding free energy between DNA_*i*_ and the protein. The difference in the binding free energy (Δ*G*_bind,2_ – Δ*G*_bind,1_) was calculated as Δ*G*(complex_1→2_) – Δ*G*(DNA_1→2_). In the present study, the binding free-energy difference was calculated between the canonical G-box motif (C_1_A_2_C_3_G_4_T_5_G_6_) and an altered sequence (T_1_A_2_C_3_G_4_T_5_G_6_ or C_1_G_2_C_3_G_4_T_5_G_6_) for each of the AtBIL1/BZR1-DNA and AtMYC2-DNA systems. Each complex and DNA-alone system was equilibrated in a 1-μs MD simulation. The final structure was used as the initial structure of the free-energy perturbation calculation. The calculations were repeated six times with different initial velocities. C_1_ or A_2_ of the first nucleotide chain was altered in the first three calculations, and C_1_ or A_2_ of the second nucleotide chain (or equivalently, G_6_ or T_5_ of the first nucleotide chain) was altered in the last three calculations. The average and standard deviation of the binding free-energy difference values obtained from the six calculations are shown.

## Supplementary Information

Supplementary Figures.
