# Supplementary material for: Highlighting the potential utility of MBP crystallization chaperone for Arabidopsis BIL1/BZR1 transcription factor-DNA complex
Source: Sci Rep. 2021 Feb 16;11:3879. doi: 10.1038/s41598-021-83532-2 (PMC7887268; doi:10.1038/s41598-021-83532-2)
Supplement: Supplementary file 1 — Supplementary Figures. [file 41598_2021_83532_MOESM1_ESM.pdf]

Supplementary Information for:

Highlighting the potential utility of MBP crystallization chaperone for *Arabidopsis* BIL1/BZR1 transcription factor-DNA complex

Shohei Nosaki[1], Tohru Terada[2], Akira Nakamura[1], Kei Hirabayashi[1], Yuqun Xu[1], Thi Bao Chau Bui[1], Takeshi Nakano[3,4], Masaru Tanokura[1,*] & Takuya Miyakawa[1,*]

[1]Department of Applied Biological Chemistry, Graduate School of Agricultural and Life Sciences, The University of Tokyo, Tokyo 113-8657, Japan

[2]Department of Biotechnology, Graduate School of Agricultural and Life Sciences, The University of Tokyo, Bunkyo-ku, Tokyo 113-8657, Japan

[3]Graduate School of Biotsudies, Kyoto University, Sakyo-ku, Kyoto, 606-8502, Japan

[4]Gene Discovery Research Group, RIKEN CSRS, Wako, Saitama, 351-0198, Japan

*Corresponding authors. Email: amtanok@mail.ecc.u-tokyo.ac.jp (M.T.) and atmiya@mail.ecc.u-tokyo.ac.jp (T.M.)

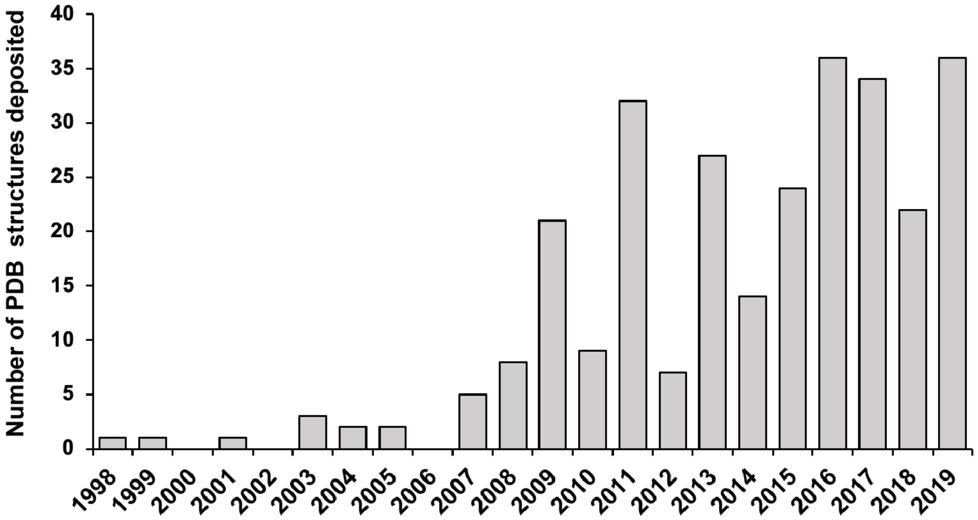

Supplementary Figure 1. Number of MBP-mediated solved structures that have been deposited in the Protein Data Bank (PDB).

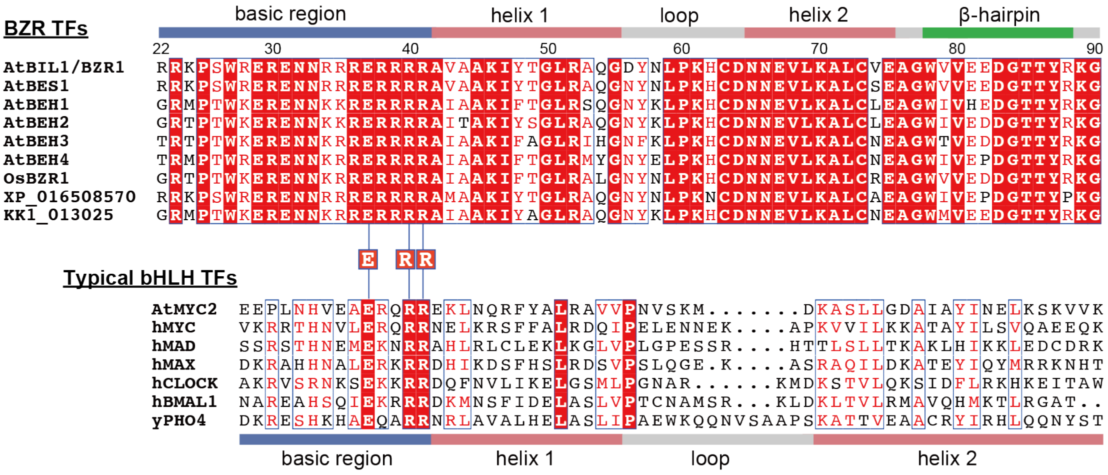

Supplementary Figure 2. Features of amino acid sequences of the BZR DBD. Amino acid sequence alignments of DBDs of BZR TFs (upper panel) and typical G-box-binding bHLH TFs (lower panel). Aligned sequences include AtBIL1/BZR1, AtBES1 and AtBEH1–4 from *Arabidopsis thaliana*, OsBZR1 (LOC_Os07g39220) from *Oryza sativa*, XP_016508570 from *Nicotiana tabacum* and KK1_013025 from *Cajanus cajan* for BZR TFs and AtMYC2 from *Arabidopsis thaliana*, MYC, MAD, MAX, BMAL1 and CLOCK from *Homo sapiens* and PHO4 from yeast for typical bHLH TFs. Blue lines connect the corresponding residues that determine the specificity for the G-box motif.

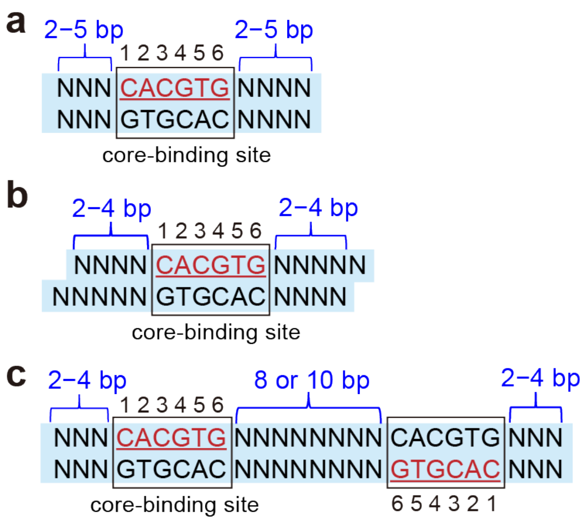

Supplementary Figure 3. DNA constructs used for cocrystallization with unfused or MBP-fused AtBIL1/BZR1. (**a**) DNA fragments with blunt ends containing a core-binding site of AtBIL1/BZR1 (the G-box motif or its variants). (**b**) DNA with one nucleotide overhanging at the 3'-ends, containing a core-binding site. (**c**) Palindromic DNA with blunt ends containing two core-binding sites.

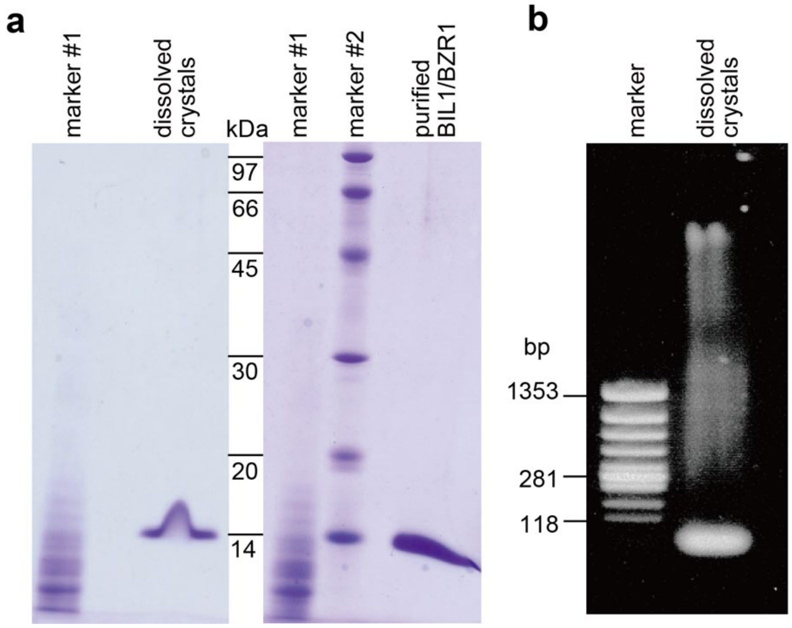

Supplementary Figure 4. Crystals contain both the unfused BIL1/BZR1 and the target DNA fragment. (**a, b**) Whole gels of Fig. 1d, e, showing SDS-PAGE analysis (**a**) and agarose gel electrophoresis analysis (**b**) of dissolved crystals. An SDS-PAGE result of the purified BIL1/BZR1 was shown for comparison. Marker #1 is a low molecular weight marker and insufficiently separated.

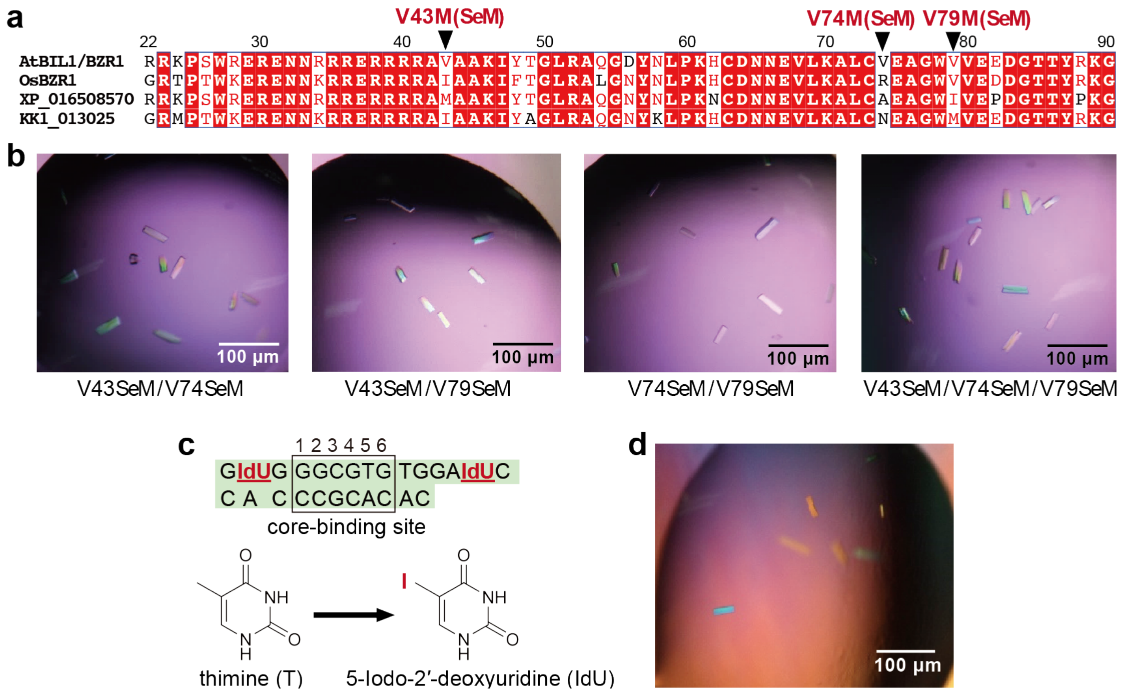

Supplementary Figure 5. Cocrystallization of the unfused AtBIL1/BZR1-DNA for phasing. (**a**) Amino acid sequence alignment of DBDs of the four BZR TFs, namely, AtBIL1/BZR1, OsBZR1, and XP_016508570 from *N. tabacum* and KK1_013025 from *C. cajan*. Three residues that were mutated to methionine for the production of selenomethionine (SeMet) derivatives are shown above the alignment. (**b**) Crystals of SeMet-containing unfused AtBIL1/BZR1 mutants in complex with nonlabeled DNA. (**c**) A crystallization construct of the DNA fragment labeled with 5-iodo-2'-deoxyuridine (IdU) as a thymine analog. (**d**) Crystals of unfused native AtBIL1/BZR1 in complex with IdU-labeled DNA.

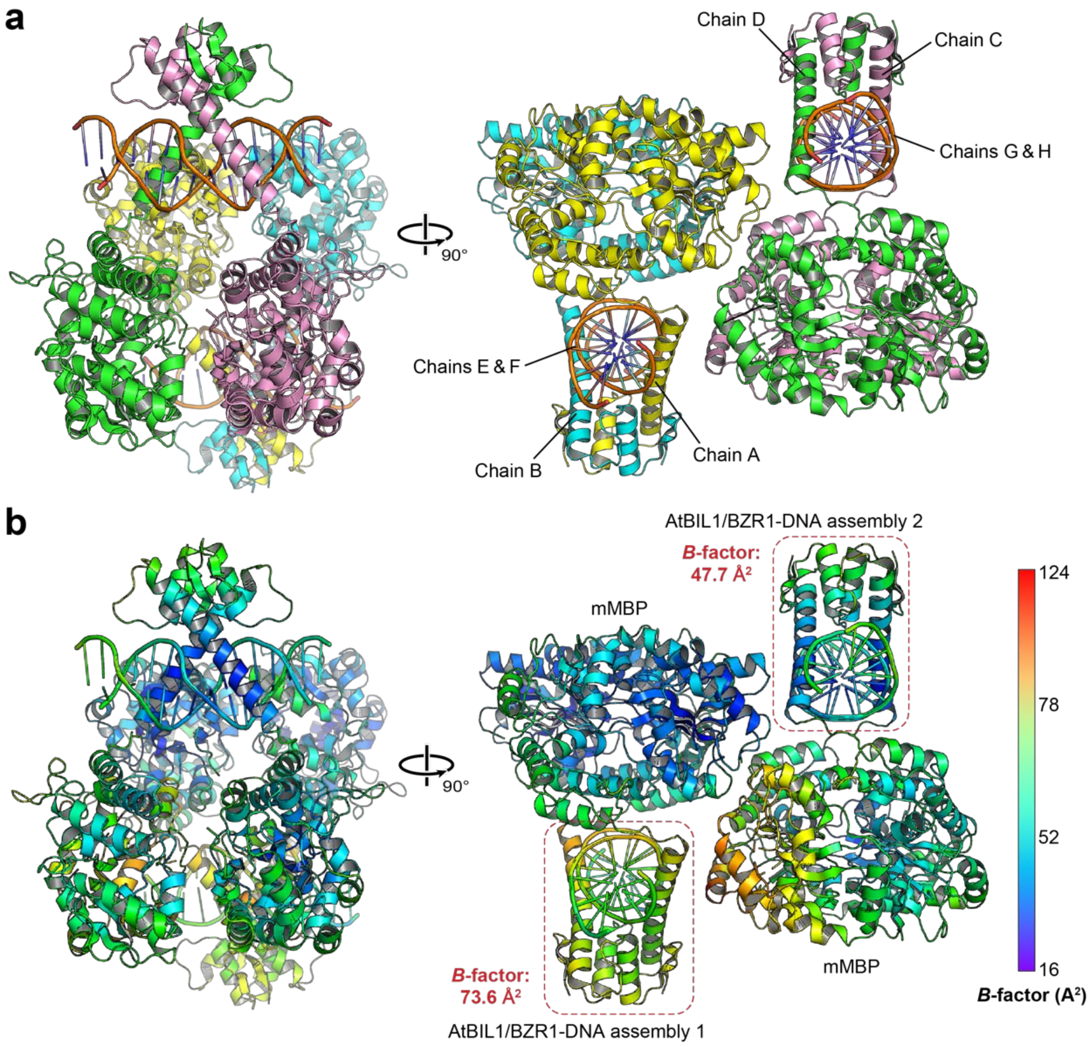

Supplementary Figure 6. Overall structure of MBP-fused AtBIL1/BZR1 in complex with DNA in the asymmetric unit. Front view (left) and side view (right) of the mMBP-Ala-AtBIL1/BZR1-DNA complex structure (PDB ID: 5ZD4) displayed as a cartoon diagram, depicted by PyMOL viewer (Version 2.3.5, Schrödinger, LLC). (**a**) Different chains of mMBP-Ala-AtBIL1/BZR1 (chains A–D) are displayed in different colors. (**b**) Representation of the complex is colored according to the *B*-factor value. Warm colors indicate high *B*-factor values, whereas cold colors indicate low *B*-factor values. The average *B*-factor value of each AtBIL1/BZR1-DNA assembly is shown.

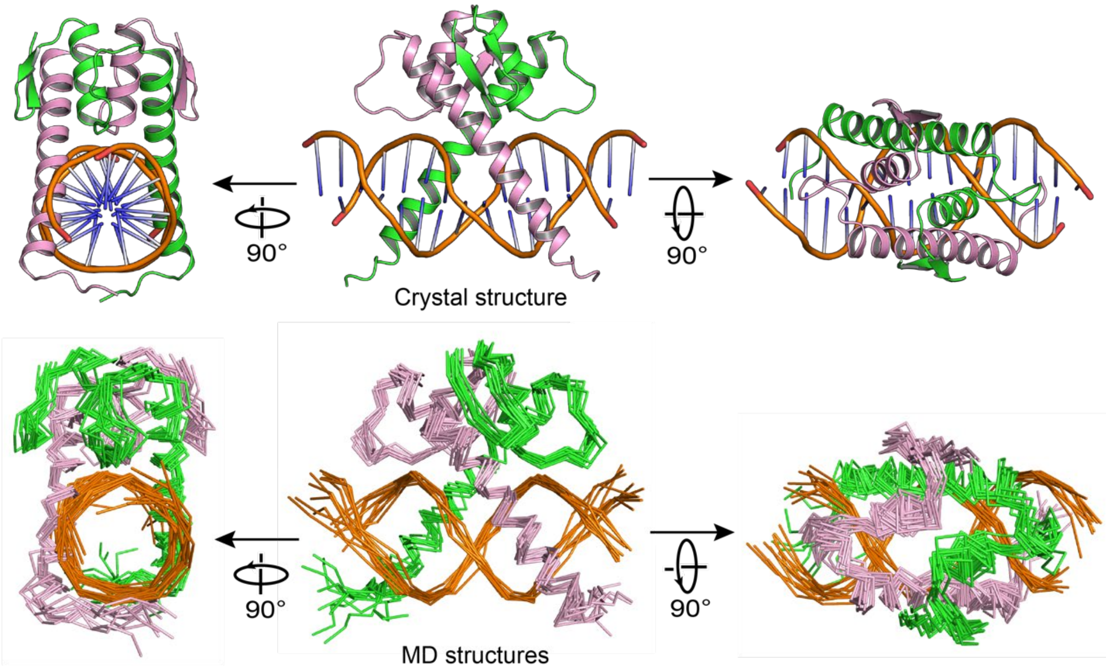

Supplementary Figure 7. The crystal structure and MD structures of AtBIL1/BZR1-DNA. Front (middle), top (left) and side (right) views of the crystal structure and MD structures (every 100 nanoseconds (ns) up to 1000 ns) of the AtBIL1/BZR1-DNA complex (PDB ID: 5ZD4, chains C, D, G and H).

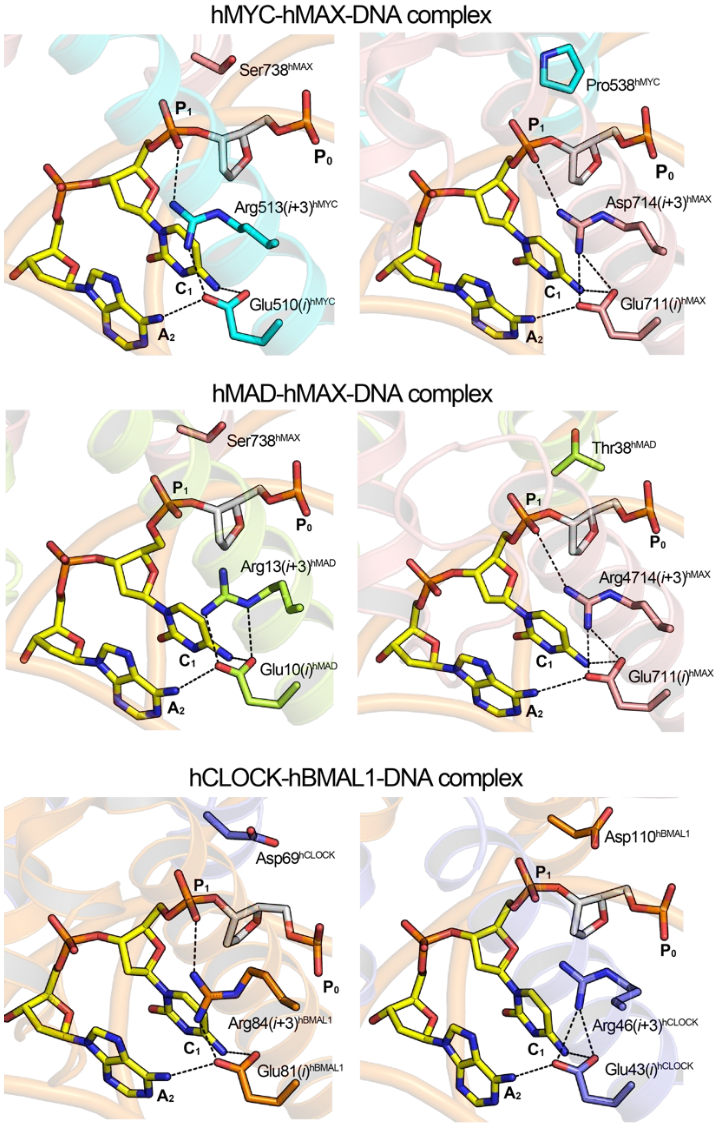

Supplementary Figure 8. The C_1A_2 base recognition mode of typical human bHLH TFs. The essential hydrogen-bonding networks for C_1A_2 base recognition by human MYC-MAX (PDB ID: 1NKP), MAD-MIX (PDB ID: 1NLW) and CLOCK-BMAL1 (PDB ID: 4H10) heterodimers. 'P_N' represents a phosphate group at position N. Hydrogen bonds (including salt bridges) are shown as dashed lines.

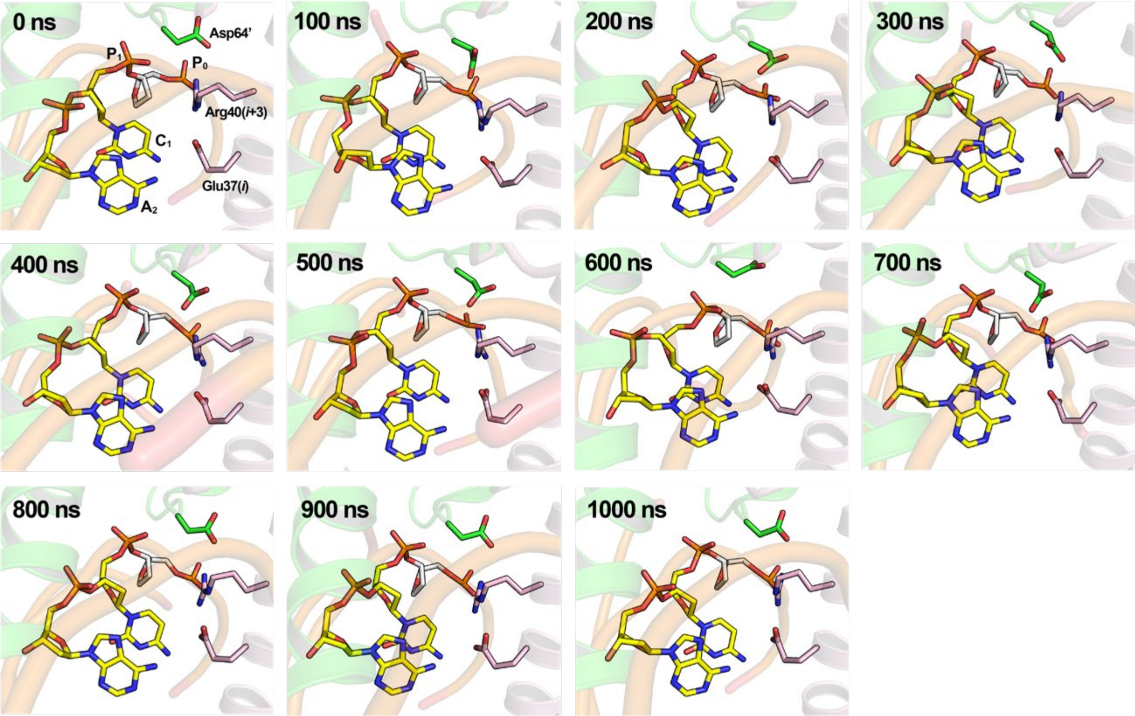

Supplementary Figure 9. Snapshots of the residues for C_1A_2 base recognition in MD simulations of the AtBIL1/BZR1-DNA complex (every 100 nanoseconds (ns) up to 1000 ns). The residues with or without a prime mark (’) belong to chains β and α of Run 3, respectively. ‘P_N’ represents a phosphate group at position N.
